# Protective Action of 3,5-Diiodo-L-Thyronine on Cigarette Smoke-Induced Mitochondrial Dysfunction in Human Alveolar Epithelial Cells

**DOI:** 10.3390/biomedicines13051014

**Published:** 2025-04-22

**Authors:** Francesca Panico, Davida Mirra, Giuseppe Petito, Giuseppe Spaziano, Vitale Del Vecchio, Renata Esposito, Rosalba Senese, Vincenzo Desiderio, Antonia Lanni, Bruno D’Agostino

**Affiliations:** 1 Department of Health Sciences, Magna Græcia University, 88100 Catanzaro, Italy; francesca.panico@studenti.unicz.it; 2 Department of Environmental Biological and Pharmaceutical Sciences and Technologies, University of Campania Luigi Vanvitelli, 81100 Caserta, Italy; davida.mirra@unicampania.it (D.M.); giuseppe.petito@unicampania.it (G.P.); giuseppe.spaziano@unicampania.it (G.S.); rosalba.senese@unicampania.it (R.S.); antonia.lanni@unicampania.it (A.L.); bruno.dagostino@unicampania.it (B.D.); 3 Department of Experimental Medicine, Histology and Embryology Section, University of Campania “L. Vanvitelli”, 80138 Naples, Italy; vitale.delvecchio@unicampania.it (V.D.V.); vincenzo.desiderio@unicampania.it (V.D.)

**Keywords:** cigarette smoke, mitochondrial dysfunction, 3,5-diiodo-L-thyronine, alveolar epithelial cells, oxidative stress

## Abstract

**Background**: Cigarette smoke (CS) is a major risk factor for chronic lung conditions. Oxidative stress and mitochondrial dysfunction play a crucial role in CS-induced pulmonary injury. 3,5-Diiodothyronine (T2) affects energy metabolism, having mitochondria as a major target. However, the underlying mechanisms of T2 related to lung diseases are poorly understood. **Aims**: To investigate the protective action of T2 on CS-induced mitochondrial dysfunction in an in vitro model of human epithelial alveolar cells. **Methods**: ATP synthesis and cytochrome c oxidase (COX) activity, as a marker of mitochondrial function, was assessed in A549 cells pretreated with T2 and exposed to CS using a bioluminescence assay and an Oroboros 2k-Oxygraph system, respectively. An evaluation of the oxidative status was conducted by assessing superoxide radical production, superoxide dismutase (SOD) activity, and H_2_O_2_ levels. Moreover, we investigated the mitochondrial mass via Mito-Tracker Green (MTG) staining and flow cytometry analysis. **Results**: CS significantly reduced ATP production. T2 pretreatment was found to prevent CS-induced impairments in ATP synthesis, enhancing COX activity. Additionally, the 2 h T2 pretreatment of CS-exposed cells mitigated CS-induced oxidative stress, thereby enhancing SOD activity and reducing the superoxide anion and H_2_O_2_ levels. Finally, MTG labeling was correlated with CS-induced mitochondrial mass gain, which is associated with cell senescence. Unexpectedly, T2 was not able to significantly prevent this mass increment, probably due to its rapid mode of action. **Conclusions**: Our results provide new insights into the protective effects of T2 against CS-induced mitochondrial damage.

## 1. Introduction

Cigarette smoke (CS) is considered a major global health concern, contributing to the onset and progression of severe lung injury, a difficult and debilitating condition often associated with chronic obstructive pulmonary disease (COPD), acute respiratory distress syndrome (ARDS), lung cancer, and asthma [1,2,3,4,5,6,7,8]. The inhalation of CS’ toxic components, such as nicotine and various stable compounds, promotes aberrant cellular processes, genetic and epigenetic changes, and alterations in sphingolipid profiles and inflammatory signaling pathways [9,10,11,12,13,14]. Although the complete spectrum of lung impairments mediated by CS remains incompletely understood, mitochondrial dysfunction is increasingly believed to play an important role in the pathophysiology of lung diseases. Mitochondria, often referred to as the metabolic powerhouses of the cell, are dynamic organelles involved in several essential cellular processes, including ATP production, calcium signaling, apoptosis, and the generation of reactive oxygen species (ROS) [15]. CS has been shown to induce mitochondrial damage in alveolar epithelial cells by altering their structural integrity and functional capacity. Specifically, CS compounds can accumulate within mitochondria, altering the mitochondrial respiratory chain and impairing cellular ATP production, which leads to the generation of toxic oxygen metabolites [16,17]. The genesis of oxidative stress is not only attributable to excessive or unregulated ROS formation but also linked to the dysregulation of the antioxidant system. Research indicates that CS exposure suppresses or downregulates antioxidant enzymes such as superoxide dismutase (SOD) and catalase in erythrocytes and plasma. Furthermore, in vitro CS exposure led to reductions in the total glutathione (GSH) levels by amplifying its consumption and repressing its synthesis or regeneration [18,19,20,21]. Additionally, the activity of cytochrome c oxidase (COX), a key component of the mitochondrial respiratory chain, has been reported to be decreased in the skeletal muscle mitochondria of smokers [22]. Currently, bronchodilators, inhaled corticosteroids, phosphodiesterase-4 (PDE4) inhibitors, mucolytic agents, and oxygen therapy are able to improve quality of life and reduce exacerbation in patients affected by CS-induced lung injury. However, not all of these drugs act on the underlying mitochondrial damage or oxidative stress, and their long-term use may lead to tolerance or severe side effects [23,24]. As current therapies provide symptomatic alleviation, it is essential to investigate new agents that effectively target the root causes of lung injury. In this scenario, 3,5,3′-triiodo-L-thyronine (T3) could improve mitochondrial function in models of lung injury, indicating a potential protective role of thyroid hormones (TH) against oxidative stress-induced mitochondrial damage [25,26,27]. THs are represented by 3,3′,5,5′-tetraiodo-L-thyronine (T4) and the biologically active T3, which is primarily generated through the deiodination of T4 in peripheral tissue [28]. THs regulate the energy metabolism of the entire body, primarily through the transcriptional activity of their nuclear receptors. Among the derivatives of THs, 3,5-diiodo-L-thyronine (T2), a biologically active metabolite of T3, has garnered attention for its physiological effects, which include roles in energy metabolism regulation, as well as exhibiting antioxidant and anti-inflammatory properties. Evidence suggests that T2 primarily exerts its effects through mechanisms that are independent of thyroid hormone receptors (THRs), with mitochondria identified as primary targets. Its effects include the stimulation of oxygen consumption, the enhancement of mitochondrial activity [29], increased ATP synthesis, and an increased cellular oxidative capacity [30]. Furthermore, it is important to note that T2 does not suppress TSH with the same intensity as T3 in animal models, implying the possibility of separating desirable from thyrotoxic effects [30,31]. However, the underlying mechanisms of T2’s action in lung injury models are poorly understood. Therefore, investigating the potential role of T2 in mitigating lung diseases is warranted. Accordingly, the present study aims to examine the molecular mechanisms by which T2 may confer protection against CS-induced mitochondrial dysfunction in an in vitro model of human epithelial alveolar cells.

## 2. Methods

### 2.1. Cell Culture and Treatment

**Figure 1:**
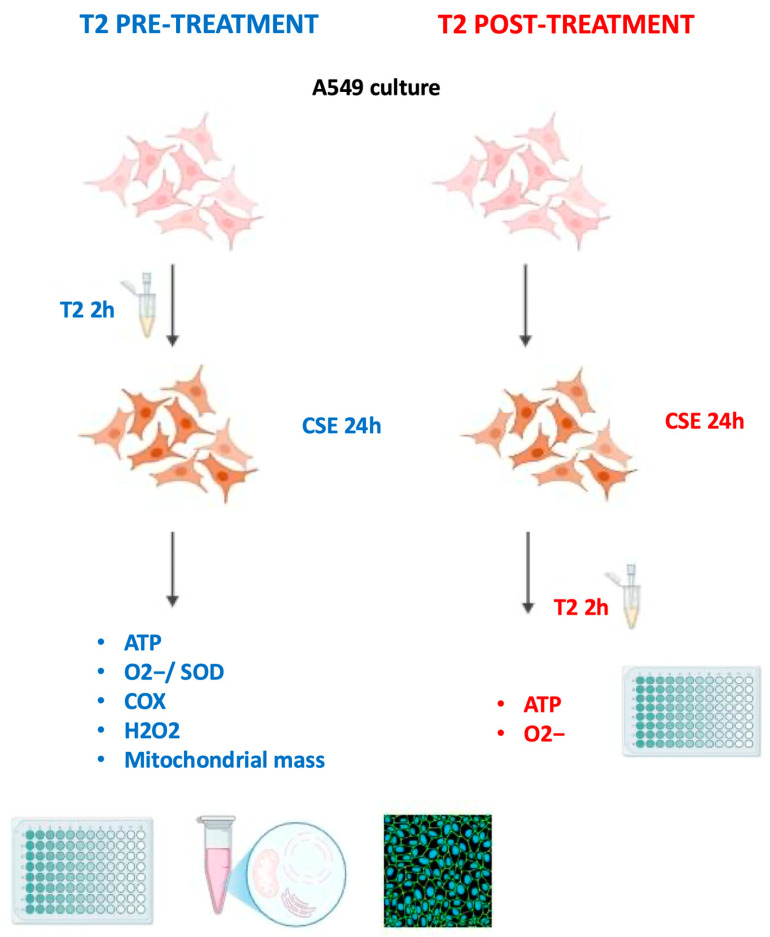
Overview of the experimental setup.

The human lung epithelial cell line A549, widely used as a model to study alveolar type II cell functions, was purchased from ATCC (ATCC^®^ CCL-185™, Manassas, VA USA). The cells were grown in 75 cm^2^ tissue culture flasks and maintained in Dulbecco’s Modified Eagle Medium/Nutrient Mixture F-12 (DMEM F12) (Gibco, Waltham, MA, USA) supplemented with 10% fetal bovine serum (FBS) (Gibco, Waltham, MA, USA) and 1% penicillin/streptomycin (Gibco, Waltham, MA, USA) at 37 °C and in 5% CO_2_/95% humidified air. Confluence was reached after 3–4 d in culture, and all experiments were performed on 5–8-day-old confluent A549 cells. The T2 compound (Merck, Darmstadt, Germany) was dissolved in 0.04 M NaOH (Sigma-Aldrich, St. Louis, MO, USA) and subsequently diluted with a 0.09% saline solution to produce a stock solution. All treatments were performed in DMEM F12 supplemented with 10% charcoal-stripped FBS. Notably, for pretreatment, T2 was removed from the cell culture after 2 h and replaced with 10% of cigarette smoke extract (CSE) medium; for post-treatment, following 24 h 10% CSE, the medium was replaced with 10 μM T2 for 2 h before starting the downstream analysis. Each evaluation of A549 cells was performed in three independent experiments and conducted using measures of blindness to avoid any detection bias. Figure 1 shows an overview of the experimental setup.

### 2.2. Preparation of Cigarette Smoke Extract

The CSE was prepared using Red Marlboro cigarettes (Phillip Morris, Cracow, Poland), as previously described [32]. The obtained medium was considered as 100% CSE. The CSE was sterile-filtrated, aliquoted and stored at −80 °C to reduce batch-to-batch variability, ensuring reproducibility across the experiments. In the CSE treatments, excluding the thiazolyl blue tetrazolium bromide assay, the cells were maintained in DMEM F12 supplemented with 10% CSE.

### 2.3. Thiazolyl Blue Tetrazolium Bromide Assay

A549 cells were used to assess the exact dose and timing required for the CSE to affect the mitochondrial reductive function. The cells were seeded at a density of 1 × 10^4^ per well in 96-well plates and allowed to attach overnight at 37 °C in 5% CO_2_/95% humidified air. The cells were then exposed to different CSE concentrations, as mentioned before, ranging from 2 to 40% for 24 or 48 h. Following treatments, the mitochondrial reductive function was determined by the thiazolyl blue tetrazolium bromide (MTT) assay (M5655, Sigma-Aldrich), useful as an indicator of cell metabolism. Briefly, 25 μL of MTT (2 mg/mL) was added to each well and incubated for 4 h. After this time, DMSO was added, and the mitochondrial reductive function was calculated as the percentage of absorbance at 570 nm of untreated cells using a microplate reader (BioTek Synergy H1 Agilent, Santa Clara, CA, USA).

### 2.4. ATP Assay

To investigate the ability of T2 and CSE to modulate the mitochondrial function of A549 cells, ATP production was assessed with an ATP Determination Kit (Thermo Fisher Scientific, Waltham, MA, USA), following the instructions of the manufacturer. The cells were seeded at a density of 1 × 10^4^ per well in 96-well plates and allowed to attach overnight at 37 °C in 5% CO_2_/95% humidified air. First, CSE’s ability to modulate mitochondrial ATP synthesis was evaluated. Then, to establish the exact dose of T2 for subsequent treatments, we evaluated ATP production in A549 cells in response to increasing concentrations of T2 ranging from 1 nM to 10 μM, following a time course from 15 min to 24 h. Based on these evaluations, and according to literature data [33,34], 10 μM and 2 h were selected as the best concentration and the optimal time for the ensuing analyses. Moreover, we decided to evaluate T2’s effects before and after CSE exposure. Cells were lysed with 25 μL of cell lysis buffer (BD Biosciences, Franklin Lakes, NJ, USA), and lysates were diluted 1:10 using the ATP Determination Kit reaction mix for a total volume of 100 μL. Luminescence was determined directly after the addition of the lysate to the reaction and quantified at 560 nm in a Junior LB 9509 Luminometer (Berthold Technologies, Bad Wildbad, Germany). The ATP concentrations in the experimental samples were calculated using an ATP standard curve. The percentage of ATP was normalized to the relative light units (RLU) of untreated cells. Experiments were performed in triplicate.

### 2.5. Mitochondrial Superoxide Radical Assay

To investigate the ability of T2 to mitigate CSE’s effects on the redox balance of A549 cells, superoxide anion species were measured with the Mitochondrial Superoxide Detection Kit (ab219943, Abcam, Cambridge, UK), as described by Yang et al. [35] and following the instructions of the manufacturer. The cells were seeded at a density of 1 × 10^4^ per well in 96-well plates and allowed to attach overnight at 37 °C in 5% CO_2_/95% humidified air. The cells were exposed to 10% CSE for 24 h and treated with 10 μM T2 for 2 h prior to or after CSE exposure. Cells were stained with 100 µL/well of the MitoROS 580 dye solution according to the instructions, and, after 45 min, excitation at 540 nm and emission at 590 nm was read with a microplate reader (BioTek Synergy H1). The relative superoxide production was normalized to the fluorescence intensity of untreated cells. Experiments were performed in triplicate.

### 2.6. Superoxide Dismutase Activity Assay

To further investigate CSE and T2’s ability to influence redox homeostasis, SOD activity was assessed using an SOD colorimetric activity assay (EIASODC, Invitrogen, Frederick, MD, USA), as in Remigante et al. [36]. The cells were seeded at a density of 1 × 10^6^ in a cell culture dish and allowed to attach overnight at 37 °C in 5% CO_2_/95% humidified air. The cells were pretreated with 10 μM T2 for 2 h and then exposed to 10% CSE for 24 h. Cell pellets were resuspended with phosphate-buffered saline (PBS) and homogenized with Dounce homogenizer for 30 s. Samples were centrifuged at 1500× *g* for 10 min at 4 °C, and an SOD activity assay was performed using the supernatant. The SOD activity (U/mL) in the experimental samples was calculated using an SOD standard curve, and the absorbance was measured at 450 nm with a microplate reader (BioTek Synergy H1).

### 2.7. Measurement of Hydrogen Peroxide in Cell Lysate Samples

To investigate the mitochondrial hydrogen peroxide levels in the absence or presence of T2 and CSE, H_2_O_2_ was measured in cell lysate samples using the Abcam kit (ab102500), as detailed by Li et al. [37]. Briefly, 2 × 10^6^ cells were harvested and washed in cold PBS 1X. Then, the cell lysates were resuspended in the assay buffer and homogenized rapidly with Dounce homogenizer, followed by centrifugation at 10,000× *g* at 4 °C for 2–5 min. The supernatant was collected for the deproteinization protocol. For deproteinization, we used 4M perchloric acid (PCA) (Carlo Erba Reagents S.r.l., Cornaredo, Italy) and ice-cold 2M KOH (Sigma-Aldrich). After deproteinization, we centrifuged the samples at 13,000× *g* for 15 min at 4 °C and collected the supernatant. Subsequently, we prepared a master mix of the reaction mix (assay buffer, the OxiRed Probe, developer solution V/horseradish peroxidase (HRP)) and added 50 µL of the reaction mix and 50 µL of the sample to each well. The samples were incubated at room temperature for 10 min protected from light, and the fluorescence was measured with a BioTek Synergy H1 microplate reader (excitation/emission (Ex/Em) = 535/587 nm).

### 2.8. Mitochondria Isolation and Determination of Cytochrome Oxidase Activity

To investigate the ability of T2 to mitigate CSE’s effects on the cellular oxidative capacity, the COX activity was measured in A549 cell mitochondria. The cells were seeded at a density of 5 × 10^5^ in a cell culture dish and allowed to attach overnight at 37 °C in 5% CO_2_/95% humidified air. At confluence, the cells were treated with 10 μM T2 for 2 h and then exposed to 10% CSE for 24 h. Cell pellets were resuspended in an isolation medium containing 1 mM ATP, 50 mM HEPES, 100 mM KCl, 5 mM MgCl_2_, 1 mM EDTA, and 5 mM EGTA (all reagents were sourced from Sigma-Aldrich) (pH 7.4) and gently homogenized with a Potter-Elvehjem homogenizer (Heidolph Instruments, Kelheim, Germany). The homogenate was centrifuged at 800× *g* for 10 min at 4 °C. Subsequently, the supernatant was separated from the pellet and centrifuged at 3000× *g* for 30 min at 4 °C. The mitochondrial pellet was then washed twice, resuspended in a minimal volume of isolation medium, and kept on ice. COX activity measurement was performed as previously described [38]. Aliquots of mitochondria were incubated for 30 min at 0 °C after the addition of 1.0 mg/mL Lubrol (Merck). COX activity was determined polarographically at 37 °C using the Oroboros 2k-Oxygraph system instrument (Oroboros Instruments, Innsbruck, Austria). The mitochondrial homogenate was incubated in 2 mL of reaction medium containing 30 μM cytochrome c, 4 μM rotenone, 0.5 mM dinitrophenol, 10 mM Na-malonate, and 75 mM HEPES at pH 7.4. After about 10 min, the substrate (4 mM Na ascorbate (with 0.3 mM N,N,N′,N′-tetramethyl-p-phenylenediamine (all reagents were sourced from Sigma-Aldrich)) was added and oxygen consumption was detected. The substrate auto-oxidation was evaluated in parallel measurements without mitochondrial homogenates. Sample protein content was determined using the Bio-Rad DC method (Bio-Rad Laboratories S.r.l., Segrate, Italy).

### 2.9. MitoTracker Green Flow Cytometry Analysis and Cell Staining

Other researchers have selected MitoTracker Green (MTG) flow cytometry analysis to measure mitochondrial mass levels [39,40]. We investigated the mitochondrial mass levels in the absence or presence of T2 and CSE by staining cells with the MTG fluorescent probe (M7514, Thermo Fisher Scientific), following the instructions of the manufacturer. The cells were seeded at a density of 5 × 10^4^ per well in 24-well plates and allowed to attach overnight at 37 °C in 5% CO_2_/95% humidified air. The cells were treated with 10 μM T2 for 2 h and then exposed 10% CSE for 24 h. Following treatments, cells were incubated with the vehicle or 150 nM MTG in DMEM F12 supplemented with 10% charcoal-stripped FBS for 45 min at 37 °C. Afterwards, the cells were trypsinized and the median MTG fluorescence intensity was measured on a FACSCantoTM II cytometer (BD Biosciences), illuminating with an Ar 488 nm laser and excluding cell debris and doublets from the analysis. Specific changes in the MTG signal intensities were quantified by subtracting the fluorescence of unstained cells and normalizing it to that of untreated cells. Experiments were performed in triplicate. In another set of experiments, the cells were seeded at a density of 5 × 10^4^ per well in 24-well plates on 18 mm coverslips, treaded with 10 μM T2 for 2 h and 10% CSE for 24 h, and then stained with 150 nM MTG as described above. After fixation with a solution of 2% paraformaldehyde, images were acquired by the EVOS M5000 Imaging System (Thermo Fisher Scientific).

### 2.10. Statistical Analysis

The statistical test used in these analyses was one-way analysis of variance followed by Tukey’s multiple comparison test. Statistical analyses were performed using the GraphPad software (version 9.0) (GraphPad Software, San Diego, CA, USA). Differences were considered statistically significant at *p* < 0.05. Data are shown as the mean ± standard deviation (SD).

## 3. Results

### 3.1. Cigarette Smoke Extract Significantly Affects Mitochondrial Reduction Properties

**Figure 2:**
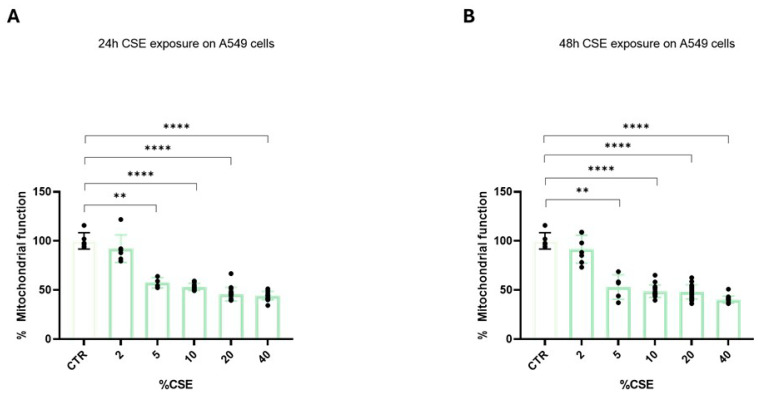
A549 cells were used to assess the exact dose and timing of CSE that was able to affect mitochondrial function. The cells were exposed to different CSE concentrations ranging from 2 to 40% for 24 h (**A**) or 48 h (**B**). Following treatments, mitochondrial function was determined by the MTT assay. The relative mitochondrial function was calculated as the percentage of absorbance at 570 nm of untreated cells. Data are representative of three independent experiments and values are expressed as mean ± SD. The statistical test used in these analyses was one-way analysis of variance followed by Tukey’s multiple comparison test. ** *p* < 0.01, **** *p* < 0.0001.

To determine the best time and dose of CSE exposure, we exposed A549 cells for 24 or 48 h to different CSE concentrations ranging from 2 to 40%. Following 24 h CSE exposure, the mitochondrial function of A549 cells was significantly affected, with the maximum reduction at a 10% concentration (Figure 2A). After this point, and with higher concentrations of CSE, we did not find a further significant decrease in mitochondrial function (Figure 2A,B). Therefore, 24 h exposure and a 10% concentration of CSE were selected to perform subsequent evaluations, being the time and dose at which the greatest mitochondrial damage occurred.

### 3.2. Mitochondrial Function and ATP Synthesis

**Figure 3:**
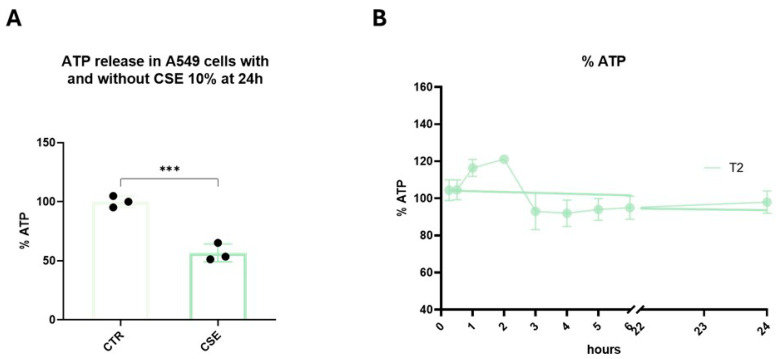
A549 cells were used to assess the ability of CSE to affect ATP production. The cells were exposed to 10% CSE for 24 h (**A**) or 10 μM T2 from 15 min to 24 h (**B**). After cell lysis, ATP production was assessed by a bioluminescence assay. The percentage of ATP was normalized to the RLU of untreated cells. Data are representative of three independent experiments and values are expressed as mean ± SD. The statistical test used in these analyses were one-way analysis of variance followed by Tukey’s multiple comparison test. *** *p* < 0.001.

We first investigated the effects of CSE on ATP production as the best determinant of mitochondrial function. As expected, CSE treatment significantly reduced mitochondrial ATP synthesis compared to the control group (*p* < 0.001) (Figure 3A). A time/concentration response curve of 15 min–24 h/1 nM–10 μM T2 was obtained. In particular, 10 μM T2 exhibited a rapid boost in ATP synthesis, with a peak reached at 2 h, showing the rapid action of the hormone. After this point, there was no further increase in mitochondrial ATP production (Figure 3B). Therefore, T2 10 μM for 2 h was selected as the best concentration and the optimal time to perform the following exposures.

### 3.3. T2 Pretreatment Prevents Cigarette Smoke-Induced ATP Impairment

**Figure 4:**
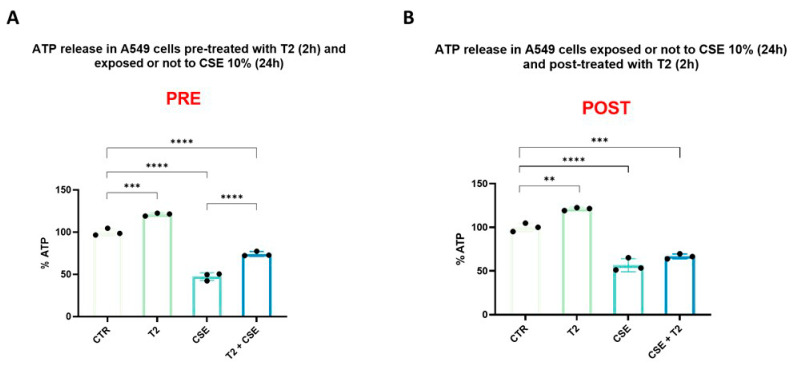
Effects of pre (**A**) and post (**B**) treatment with T2 10 μM for 2 h on stimulation with CSE. After cell lysis, ATP production was assessed by a bioluminescence assay. The percentage of ATP was normalized to the RLU of untreated cells. Data are representative of three independent experiments and values are expressed as mean ± SD. The statistical test used in these analyses was one-way analysis of variance followed by Tukey’s multiple comparison test. ** *p* < 0.01, *** *p* < 0.001, **** *p* < 0.0001.

We tested the ability of T2 to modulate mitochondrial ATP synthesis before and after CSE exposure. CSE caused a 50% reduction in ATP production (*p* < 0.0001) (Figure 4A,B), while T2 treatment enhanced ATP synthesis by 20% (*p* < 0.001) (Figure 4A), resulting in a significantly lower impact of the subsequent CSE exposure on the overall ATP levels (*p* < 0.0001) (Figure 4A). Conversely, T2 treatment after CSE exposure did not affect ATP synthesis, suggesting the inability to counteract CSE damage once established (Figure 4B).

### 3.4. T2 Pretreatment Mitigates Cigarette Smoke-Induced Effects on Cellular Oxidative Capacity

**Figure 5:**
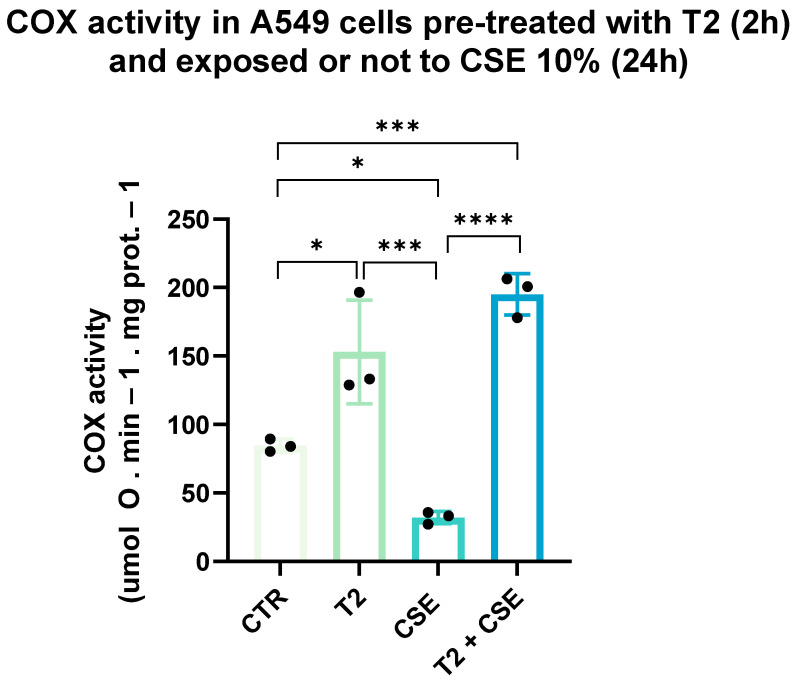
A549 cells were used to assess the ability of T2 to increase COX activity. The cells were pretreated with 10 µM T2 for 2 h and then exposed to CSE. COX activity was measured using the Oroboros 2k-Oxygraph system. Data are representative of three independent experiments and values are expressed as mean ± SD. The statistical test used in these analyses was one-way analysis of variance followed by Tukey’s multiple comparison test. * *p* < 0.05, *** *p* < 0.001, **** *p* < 0.0001.

To investigate the ability of T2 to influence the cellular oxidative capacity, COX activity was assessed in A549 cell mitochondria. As shown in Figure 5, CSE significantly reduced the enzyme function compared to the control group (*p* < 0.05). In contrast, T2 treatment resulted in enhanced COX activity (*p* < 0.05), which led to a significantly lower impact of subsequent CSE exposure (*p* < 0.0001).

### 3.5. T2 Pretreatment Prevents Cigarette Smoke-Induced Oxidative Imbalance and Antioxidant Defense Impairment

**Figure 6:**
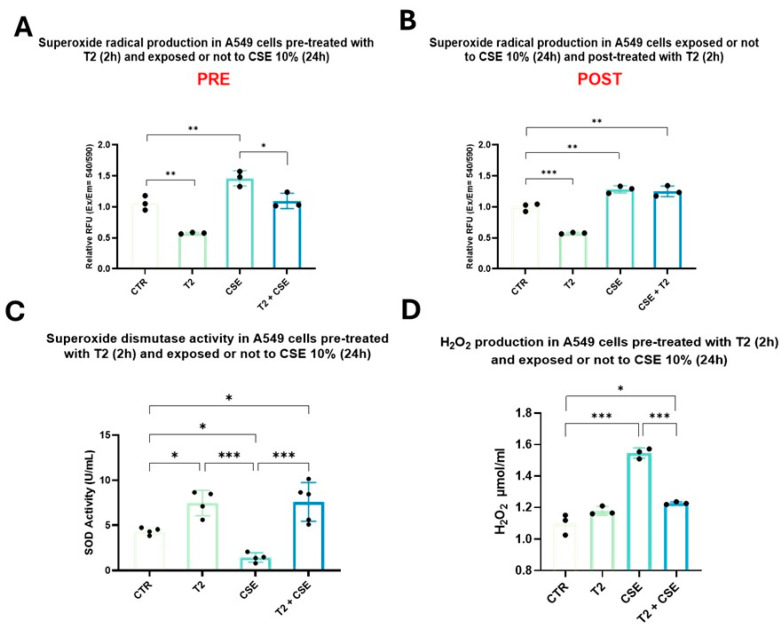
A549 cells were used to assess the ability of T2 to mitigate CSE’s effects on superoxide anion production, SOD activity, and H_2_O_2_ levels. The cells were treated with T2 pre (**A**) and post (**B**) exposure to CSE. Superoxide anion production was assessed by a fluorometric assay. The relative superoxide production was normalized to the fluorescence intensity (Ex/Em = 540/590 nm) of untreated cells. The cells were pretreated with 10 μM T2 for 2 h and then exposed to CSE (**C**,**D**). SOD activity was assessed by a colorimetric activity assay (**C**). H_2_O_2_ was assessed by a colorimetric and fluorimetric assay. Fluorescence was measured using a microplate reader (Ex/Em = 535/587 nm) (**D**). Data are representative of three independent experiments and values are expressed as mean ± SD. The statistical test used in these analyses was one-way analysis of variance followed by Tukey’s multiple comparison test. * *p* < 0.05, ** *p* < 0.01, *** *p* < 0.001.

Superoxide anion species, SOD activity, and H_2_O_2_ levels as a marker of redox homeostasis were assessed in A549 cells. CSE exposure significantly affected the oxidative status, leading to a 20% increase in the superoxide anion levels (*p* < 0.01) (Figure 6A). Conversely, T2 treatment was able to reduce the basal levels of the radical by 40% (*p* < 0.01) (Figure 6A), resulting in a significantly weaker effect on the redox status of the subsequent CSE exposure (*p* < 0.05) (Figure 6A). As expected, T2 treatment after CSE exposure did not significantly change the superoxide levels, highlighting the inability of T2 to restore the pre-existing CSE-induced oxidative imbalance (Figure 6B). Based on these results, T2 pretreatment was selected for further evaluation regarding SOD activity and H_2_O_2_ levels. CSE significantly reduced SOD activity (*p* < 0.05), while T2 pretreatment, by enhancing SOD’s basal activity (*p* < 0.05), contributed to the observed superoxide radical species decrease (Figure 6C). A significant increase in mitochondrial H_2_O_2_ levels was observed following CSE exposure compared to the control group (*p* < 0.001) (Figure 6D). Although T2 itself had no effect on the H_2_O_2_ levels, T2 pretreatment showed significant ROS scavenging activity by counteracting the CSE-induced effects on the H_2_O_2_ levels (*p* < 0.001) (Figure 6D).

### 3.6. T2 Effects on Cigarette Smoke-Induced Increment in Mitochondrial Mass

**Figure 7:**
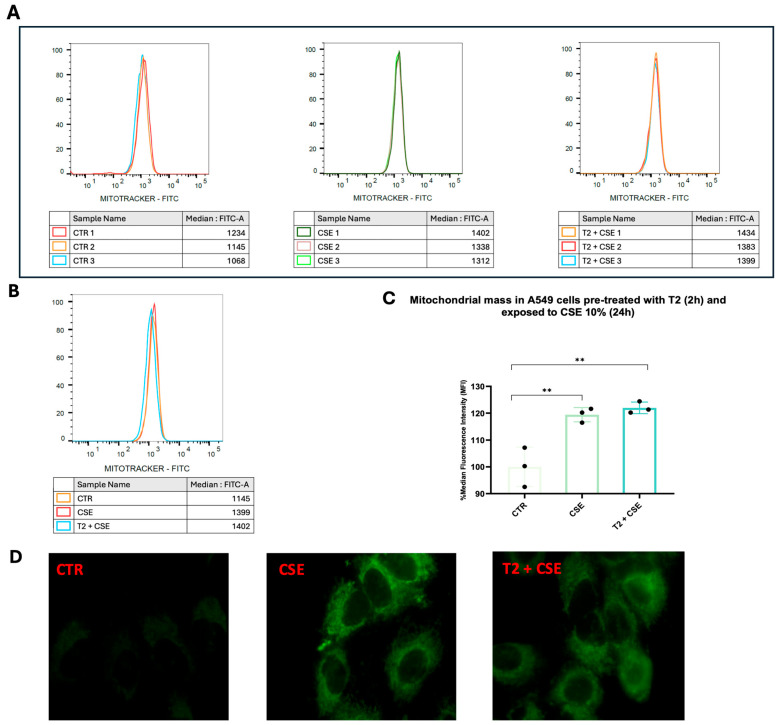
Mitochondria labeling with MTG. The cells were pretreated with T2 10 μM for 2 h and exposed to CSE. After staining with 150 nM of MTG for 45 min, cells were detached, and fluorescence was assessed with flow cytometry. MTG fluorescence signal for each group (**A**) and merged (**B**). The relative median fluorescence intensity was obtained by subtracting the fluorescence of unstained cells and normalizing to that of untreated cells (**C**). Representative images of MTG-labeled mitochondria (**D**). Cell fluorescence images were acquired by the EVOS M5000 Imaging System. Data are representative of three independent experiments and values are expressed as mean ± SD. The statistical test used in these analyses was one-way analysis of variance followed by Tukey’s multiple comparison test. ** *p* < 0.01.

We investigated the ability of CSE and T2 to influence the mitochondrial mass, whose changes are associated with cell senescence, through MTG staining. The flow cytometry analysis of MTG-labeled mitochondria showed a significantly higher median intensity of fluorescence in cells exposed to CSE than the control group, highlighting the ability of CSE to induce an increment in mitochondrial mass (Figure 7A–C) (*p* < 0.01). Otherwise, we did not find significant changes in fluorescence emission following T2 pretreatment (Figure 7A–C). Fluorescence imaging confirmed the mitochondrial mass augmentation due to CSE, as well as indicating T2 pretreatment’s inability to prevent CSE-induced mitochondrial mass gain (Figure 7D).

## 4. Discussion

Here, we demonstrate, for the first time, the properties of T2 in preventing CS-induced mitochondrial dysfunction in an in vitro model of human epithelial alveolar cells. CS is widely recognized as a primary risk factor for several lung diseases, leading to chronic inflammation, structural damage, pulmonary cell apoptosis, and senescence. Within this context, mitochondria play a crucial role based on their high susceptibility to the deleterious effects of CS. Indeed, components of CS can accumulate within mitochondria, inducing damage and impairing the mitochondrial respiratory chain [16,17]. Mitochondrial dysfunction can lead, in turn, to inflammatory cell infiltration, cytokine release, microvascular hyperpermeability, the release of mitochondrial damage-associated molecular patterns (mtDAMPs), ROS production, and mitochondrial DNA damage, which are key hallmarks of lung injury [41]. Mitochondrial function and fitness are generally regulated by THs, which support basal energy expenditure primarily through their effects on carbohydrate and lipid catabolism. Moreover, THs, mainly T3, could play a crucial role in lung development and function, mitigating lung injury and preserving mitochondrial integrity and oxidative balance [26,42]. Given the importance of mitochondrial dysfunction as a hallmark of lung injury, T2, a biologically active metabolite of T3 that binds rapidly and directly to mitochondria, could represent a potential therapeutic tool in lung injury models. Indeed, T2 could mitigate cellular damage in lung tissue following injurious stimuli, such as CS, by enhancing mitochondrial respiration and reducing oxidative stress. Overall, the underlying mechanisms of T2’s action are not fully understood, and its effects in the context of lung diseases have been poorly investigated. Therefore, we aimed to elucidate the effects of T2 using an in vitro model of CS-induced mitochondrial dysfunction. Our data showed that in vitro exposure to CS significantly reduced mitochondrial function and ATP generation, which is a critical determinant of mitochondrial integrity due to its crucial role in maintaining cellular metabolic functions. ATP synthesis occurs through a multi-step process carried out by five different protein complexes via the phosphorylation of ADP [43]. Consistent with our findings, van der Toorn et al. demonstrated that CS can directly inhibit the mitochondrial respiratory chain, resulting in increased oxidant levels, impaired ATP production, and diminished mitochondrial respiration [44]. Moreover, CS was found to promote a reduction in mitochondrial membrane potential and a decline in ATP biosynthesis by activating the adenine nucleotide translocator (ANT) and increasing the mitochondrial membrane permeability (MMP) [45]. Interestingly, T2 pretreatment effectively prevented CS-induced impairments in energy generation, whereas T2 treatment following CS exposure was unable to counteract the damage caused by CS. The effectiveness of pretreatment appeared to be primarily due to an increase in ATP synthesis, which resulted in a significantly lower impact of subsequent CSE exposure on the overall ATP levels. Currently, there is limited literature on the potential effects of T2 in lung diseases; however, some protective effects of TH pretreatment, particularly T3, on impaired lung function have been reported. For instance, Zhang et al. [26] demonstrated that T3 pretreatment enhanced the mitochondrial anti-ROS potential, improved biogenesis and mitophagy, and attenuated apoptosis via PTEN-induced kinase I (PINK1) in an in vivo model of hyperoxia-induced lung injury. Furthermore, different authors have highlighted that both hyperthyroidism and the combination of T3 and dexamethasone pretreatment can improve alveolar fluid clearance and lung compliance in vivo, suggesting that thyroid function significantly influences lung mechanics [27]. The protective effect of T2 was observed within a short time interval of 2 h, indicating that the hormone may act through a rapid and transient mechanism. Consistent with our data, studies on rats and human mononuclear blood cells have shown that T2 exerts rapid effects on mitochondrial respiration, as early as 1 h after administration, highlighting its ability to stimulate cellular respiration via pathways involving direct interaction with mitochondria [46]. COX, also known as complex IV of the mitochondrial respiratory chain, plays an important role in cellular respiration by facilitating the transfer of electrons from cytochrome c to molecular oxygen, ultimately contributing to ATP production through oxidative phosphorylation [47]. Several authors have demonstrated that T2 can stimulate mitochondrial respiratory activities independently of protein synthesis and can directly bind to the Va subunit of the COX complex, suggesting that this subunit is one of the sites through which T2 exerts its effects on mitochondria [48]. Accordingly, our data showed that T2 treatment significantly enhanced COX activity, promoting mitochondrial function and leading to the observed increase in ATP synthesis. This enzyme is crucial in maintaining cellular energy homeostasis and is sensitive to various environmental factors. Different authors have suggested COX as a cellular target of CS, reporting a decrease in enzyme activity in peripheral blood mononuclear cells from acute smokers [49]. Additionally, Song et al. [50] found decreased COX subunit II mRNA and protein levels, as well as reduced COX activity, in CS-treated human umbilical vein endothelial cells (HUVECs). Our model corroborated these findings, demonstrating that CS exposure significantly reduced COX activity, which in turn contributed to the overall decrease in ATP production. However, the enhancement of COX activity induced by T2 pretreatment resulted in a significantly weaker effect on CS-induced enzyme impairment. Our results support the hypothesis that T2 pretreatment is effective in mitochondrial protection by directly and rapidly enhancing basal COX function, thus preparing cells to better sustain CS-induced damage. Moreover, we hypothesize that the weaker impact of CSE exposure on COX activity could be related to a reduction in oxidative stress and to an increase in antioxidant machinery activity. Overall, the molecular mechanism by which pre-exposure to T2 could attenuate the effects of CSE exposure on COX activity needs to be clarified. Superoxide and hydroxyl radicals, along with non-radical oxygen species such as H_2_O_2_, are collectively referred to as ROS and are generated during normal metabolic processes [51]. The damage caused by ROS results in an imbalance between these toxic molecules and antioxidant enzymatic defense mechanisms [52]. Certain chemicals present in tobacco smoke have been suggested to interfere with electron transfer, leading to impaired respiratory function and increased ROS production [49]. Indeed, it has been shown that smokers have increased numbers of alveolar macrophages, which release high levels of superoxide [53]. Accordingly, our data indicated that in vitro exposure to CS induced a significant increase in the superoxide anion levels, establishing an oxidatively imbalanced environment. On the other hand, the administration of T2 results in a significant reduction in superoxide radicals, thus counteracting the increase prompted by CS and enhancing mitochondrial protection. Antioxidant enzymes play a key role in scavenging free radicals and converting them to less harmful molecules [54]. Among the most well-known ones, SOD constitutes a major protective mechanism against ROS at the mitochondria. Findings from murine models and cellular studies have revealed that SOD activity is reduced in alveolar macrophages, erythrocytes, circulating progenitor cells, and the blood serum of smokers [55]. Consistent with this evidence, our data indicate that in vitro exposure to CS induces a significant reduction in SOD activity, which results in compromised antioxidant defense. Conversely, pretreatment with T2 mitigated this impairment due to an increase in enzyme activity, justifying the observed lower superoxide levels and supporting the hypothesis of its role as a promoter of free radical scavenging. These findings are supported by our previous evidence of higher SOD levels in liver tissue from rats receiving a daily injection of T2 for one week compared to control rats [56], as well as those of Guerra et al. [57], who found that patients with hyperthyroidism exhibited increased malondialdehyde (MDA) content and SOD activity compared to the control group. It is well known that quinone, hydroquinone, and semiquinone, produced from tobacco combustion, reduce oxygen to superoxide, which can dismutate to H_2_O_2_ and then undergo reduction to oxygen and water by catalase (CAT) in the presence of iron [58]. Similarly, glutathione peroxidase (GPX) scavenges and detoxifies H_2_O_2_, preventing oxidative stress, DNA oxidation/damage, and the consequent mutagenesis and apoptosis induced by H_2_O_2_ excess [59]. Tanni et al. [60] found that the monocyte secretion of H_2_O_2_ was statistically higher in smokers compared to healthy subjects. Moreover, nicotine has been shown to inhibit CAT and GPX activity, contributing to the accumulation of H_2_O_2_ [61]. Therefore, it is not surprising that the increased production of superoxide radicals induced by CSE can result in higher H_2_O_2_ levels. Interestingly, although T2 pretreatment induced an increase in SOD activity, the H_2_O_2_ levels were reduced, suggesting a possible role in regulating the CAT and GPX pathways to enhance the processing of CSE-derived H_2_O_2_. Currently, there are conflicting results regarding the ability of THs to modulate antioxidant enzymes’ activity [62,63]. Indeed, THs can stimulate free radical formation in the mitochondria by affecting oxygen metabolism; however, this mechanism could also lead to an enhancement in intracellular scavenging enzymes such as CAT and GPX. Moreover, it is important to emphasize that the response to iodothyronine is tissue- and hormone-dependent and that T2’s effects are not directly linked to other THs. Therefore, the literature data cannot be generalized to T2. A recent review gathered data on CS’ ability to activate mitophagy through different pathways, leading to cellular phenotypic alterations that are consistent with cellular senescence. In this context, the mitochondrial mass is considered a well-established hallmark of aging [64]. Indeed, mitochondria often increase in size and volume during senescence, both in vitro and in vivo [64]. However, because mitophagy activity is reduced in senescent cells [65], dysfunctional mitochondria accumulate, and the increase in mitochondrial mass may only partially counteract their dysfunction. Therefore, we investigated the ability of T2 before CSE exposure to influence the mitochondrial mass by MTG staining. In agreement with this, our data indicate that CS significantly increased the mitochondrial mass, confirming the ability of CSE to accelerate cellular aging and mitochondrial structural changes. Although T2 pretreatment showed protective effects on the abovementioned mitochondrial markers, unexpectedly, it was not able to prevent a CS-induced gain in mitochondrial mass. We hypothesized that the lack of T2’s effect on the mitochondrial mass before CSE exposure could be attributed to its rapid mode of action. Although we did not analyze the effects of T2 alone on the mitochondrial mass, and there is no other direct evidence of T2’s effects on the mitochondrial mass, Menzies et al. [66] showed the ability of T3 to decrease ROS production, increase COX activity, and elevate the ATP levels in primary human fibroblasts without altering the mitochondrial mass. Overall, our results provide, for the first time, insights into the metabolic role of T2 in the lungs by showing its efficacy in preventing CS-induced mitochondrial damage and oxidative stress. Here, we focused on in vitro models, which are unable to fully replicate the complexity of biological systems. Although several studies have shown the beneficial and adverse effects of T2 in different animal models [67,68,69,70], little has been reported in clinical studies [69]. In conclusion, although we did not evaluate long-term CS exposure or the in vivo effects of T2 administration, our findings could pave the way for future studies focusing on the unique biological properties of T2 and its role in smoking-related lung disease treatment.

## Data Availability

The original contributions presented in this study are included in the article. Further inquiries can be directed to the corresponding author.
